# Identifying indicators of apple bud dormancy status by exposure to artificial forcing conditions

**DOI:** 10.1093/treephys/tpae112

**Published:** 2024-08-31

**Authors:** Anton Milyaev, Ute Born, Elke Sprich, Michael Hagemann, Henryk Flachowsky, Eike Luedeling

**Affiliations:** University of Bonn, Horticultural Sciences, Institute of Crop Science and Resource Conservation (INRES), Auf dem Hügel 6, 53121, Bonn, Germany; University of Hohenheim, Institute of Crop Science, Section Production Systems of Specialty Crops (340f), Emil-Wolff-Street 25, 70599 Stuttgart, Germany; University of Hohenheim, Institute of Crop Science, Section Production Systems of Specialty Crops (340f), Emil-Wolff-Street 25, 70599 Stuttgart, Germany; University of Hohenheim, Institute of Crop Science, Section Production Systems of Specialty Crops (340f), Emil-Wolff-Street 25, 70599 Stuttgart, Germany; University of Hohenheim, Institute of Crop Science, Section Production Systems of Specialty Crops (340f), Emil-Wolff-Street 25, 70599 Stuttgart, Germany; Julius Kühn Institut (JKI) – Institute for Breeding Research on Fruit Crops, Federal Research Centre for Cultivated Plants, Pillnitzer Platz 3a, 01326 Dresden, Germany; University of Bonn, Horticultural Sciences, Institute of Crop Science and Resource Conservation (INRES), Auf dem Hügel 6, 53121, Bonn, Germany

**Keywords:** budbreak synchrony, growth chamber experiment, percentage of budbreak, Tabuenca’s test, water content in buds, winter rest

## Abstract

Dormancy in temperate fruit trees is a mechanism of temporary growth suspension, which is vital for tree survival during winter. Studies on this phenomenon frequently employ scientific methods that aim to detect the timing of dormancy release. Dormancy release occurs when trees have been exposed to sufficient chill, allowing them to resume growth under conducive conditions. This study investigates dormancy dynamics in two apple (*Malus × domestica* Borkh.) cultivars, ‘Nicoter’ and ‘Topaz’, by sampling branches in an orchard over 14 weeks (2019 to 2020) and over 31 weeks (2021 to 2022) and subjecting them to a 42-day budbreak forcing period in a growth chamber. Temporal changes in budbreak percentages demonstrated dormancy progression in the studied apple cultivars and allowed the three main dormancy phases to be distinguished: paradormancy (summer dormancy), endodormancy (deep dormancy) and ecodormancy (spring dormancy), along with transition periods between them. Using these data, we explored the suitability of several alternative methods to determine endodormancy release. Tabuenca’s test, which predicts dormancy release based on the differences in dry weights of buds with and without forcing, showed promise for this purpose. However, our data indicated a need for considerable adjustments and validation of this test. Bud weight and water content of buds in the orchard did not align with budbreak percentages under forcing conditions, rendering them unsuitable for determining endodormancy release in ‘Nicoter’ and ‘Topaz’. Shoot growth cessation did not seem to be connected with either dormancy progression or dormancy depth of the studied cultivars, whereas leaf fall coincided with the beginning of the transition from endo- to ecodormancy. This work addresses methodological limitations in dormancy research and suggests considering the mean time to budbreak and budbreak synchrony as additional criteria to assess tree dormancy status.

## Introduction

Many tree species exploit a physiological and genetic mechanism of temporary growth suspension during periods of adverse climatic conditions to avoid temperature injuries of susceptible plant organs, primarily bud meristems. Such a protection mechanism is known as dormancy (Doorenbos 1953). Temperate fruit trees, including apple (*Malus* × *domestica* Borkh.), undergo a period of dormancy to survive unfavorable conditions during winter (Faust et al. 1997). When dormant, trees do not show any visible growth and normally resume their developmental cycle in the following spring, after the environmental conditions have become conducive to growth resumption. This mechanism of dormancy, which many tree species developed through a long process of evolution and which decisively determines the time of flowering, has been attracting scientific attention for at least two centuries (Saure 1985; Campoy et al. 2011*a*). However, it is still unclear which molecular signals initiate and terminate dormancy in tree species and how the trees use environmental cues to regulate the timing of dormancy onset and dormancy release (Beauvieux et al. 2018; Ding et al. 2024).

Dormancy in fruit trees is a dynamic process that is commonly divided into three phases: para-, endo- and ecodormancy (Lang et al. 1987). During paradormancy (summer dormancy), budbreak can still be stimulated by specific orchard management practices, such as defoliation and the application of dormancy-breaking agents (Edwards 1987; Erez 2000), if the ambient temperatures are still favorable for growth. As soon as the ambient temperature decreases (at the end of the growing season), the trees enter the next phase of dormancy, which is referred to as endodormancy (deep dormancy), or winter dormancy (Heide and Prestrud 2005). Several studies have indicated that growth cessation and onset of dormancy are temperature-dependent processes (Tanino et al. 2010), which might share some common genetic background (Moser et al. 2020). There is, however, no clear evidence as to whether shoot growth cessation and the onset of bud dormancy are interconnected processes or whether they occur independently of each other.

The only known factor that effectively breaks endodormancy is exposure of the trees to a sufficient amount of chill during winter (Shaltout and Unrath 1983; Erez et al. 1988; Kaufmann and Blanke 2019). The time when the trees’ chilling requirements are fulfilled and normal growth can be resumed is known as endodormancy release, or the beginning of the ecodormancy phase (Lang et al. 1987). By the stage of ecodormancy, the trees have already received enough chill and from this point on they primarily rely on forcing (warm) temperatures. The combined effects of temperature conditions during the forcing period and during the preceding chilling period are believed to determine the timing of budbreak (Luedeling et al. 2021). Endodormancy release cannot be determined by visual inspection of buds in the orchard, since no phenological changes occur during this time. A widely used method to determine chilling requirements and hence (endo-)dormancy release in fruit trees is the exposure of single-node cuttings (Campoy et al.  2011*b*), 1-year-old shoots (Lempe et al. 2022), detached branches (Campoy et al. 2019) or whole trees (Naor et al. 2003) to artificial forcing in a growth chamber. Under forcing conditions, which usually feature temperatures between 20 °C and 25 °C, the percentage of buds that reach the stage of budbreak within a defined forcing period is used to derive information on the dormancy status of fruit trees. Attainment of a particular budbreak percentage after the prescribed period is used to determine chilling requirements and to detect endodormancy release of the trees (Okie and Blackburn 2011).

Another method that has been proposed to determine dormancy release in apricot, peach, pear (Tabuenca 1964) and apple (Malagi et al. 2015) is Tabuenca’s test. For this test, 1-year-old shoots are sampled in the orchard throughout the dormancy period. Immediately after sampling, flower buds or mixed buds (for stone fruit or pome fruit tree species, respectively) of several shoots are (freeze-)dried to obtain the dry weight (DW), whereas another portion of shoots is placed in water and kept in a growth chamber for 7 to 10 days of forcing. After the period of forcing, the DW of the remaining flower buds or mixed buds is also determined and compared with the DW of the buds without forcing. The difference in bud weights between the two conditions is expected to indicate the timing of dormancy release (El Yaacoubi et al. 2016). This test is easy to conduct, however there is little evidence as to whether it can substitute for experiments that determine the dormancy status by monitoring budbreak percentages. Moreover, to our knowledge, there have been no studies that used the same material (e.g. terminal buds) to compare budbreak percentages under artificial forcing with Tabuenca’s test, including the original paper that first described this test (Tabuenca 1964).

Experiments with budbreak forcing conditions require suitable growth chambers, which are costly and not widely available to researchers. This implies a need for alternative methods that are as reliable in determining chilling requirements as the application of artificial forcing. The water content of buds and bud weight are easily measurable with standard laboratory equipment. However, it remains unclear whether these parameters can be used as reliable indicators of the dormancy status of deciduous trees (Hsiang et al. 2021; Walde et al. 2024).

The current work aimed to study dormancy progression in two apple cultivars, ‘Nicoter’ and ‘Topaz’, during the whole period of dormancy. This was achieved by frequent sampling (weekly) of apple branches with three bud types (spur, terminal and lateral buds) and subsequent exposure of these branches to a 42-day forcing period. This experiment allowed generation of a comprehensive dataset on budbreak percentages, the mean time of budbreak and budbreak synchrony, which were calculated for each bud type after exposure of detached apple branches to artificial forcing. We used these data to address the following hypotheses. (i) Measurements of water content in buds and/or bud weight can substitute for the growth chamber experiments that are commonly used to determine the dormancy status and the chilling requirements of fruit trees. (ii) Tabuenca’s test can be applied to determine the timing of endodormancy release in apple. (iii) Shoot growth cessation and leaf fall mark a particular stage of dormancy in apple, for example the onset of endodormancy.

## Materials and methods

### Experimental orchard, plant material and budbreak forcing conditions

Plant material was obtained from 14-year-old apple trees, cultivars ‘Nicoter’ (Kanzi®) and ‘Topaz’, grafted on ‘M.9’ rootstocks. The trees were grown in an experimental orchard of the University of Hohenheim (48°42′44.3″N 9°11′33.5″E), Stuttgart, Germany. The trees were planted at 3 m × 1 m spacing, pruned as thin spindles and trained according to the common local practices for high-density orchards.

In order to distinguish para-, endo- and ecodormancy, 2- to 3-year-old horizontally oriented apple branches were cut from each cultivar at weekly intervals between 26 November 2019 and 3 March 2020 and between 16 June 2021 and 9 March 2022. Each branch was cut from a different tree. On each branch, we distinguished between spurs (short shoots of ≤ 5 cm in length) and shoots of > 5 cm in length with one pronounced terminal bud and multiple lateral buds. In commercial apple orchards, the majority of spur and terminal buds flower in spring. These buds are of major importance for crop load. This was the reason for considering them together (spur + terminal buds) in some tests. In apple, all flower buds (including lateral buds) contain not only flower structures but also leaf primordia. Such buds are referred to as ‘mixed’ buds. However, for simplicity we will use the term ‘flower buds’ for buds containing flower and leaf primordia and ‘vegetative buds’ for buds that contain leaf primordia only.

The sample size per cultivar and per sampling time point was four to eight branches with a minimum number of 30 terminal + spur buds on all branches together. After being cut from the trees, the branches were defoliated (if leaves were present), placed in buckets filled with tap water and immediately transferred to a growth chamber (Plant Climatics, Wertingen, Germany). Spur, terminal and lateral buds were counted on each branch (spur + terminal buds in 2019 to 2020 and spur, terminal and lateral buds separately in 2021 to 2022; detailed results are provided in Table S1 available as Supplementary data at *Tree Physiology* Online). In the growth chamber, the branches were exposed to budbreak forcing conditions: constant temperature of 23.5 °C and a light regime corresponding to 16-h days and 8-h nights (light intensity of 700 µmol m^–2^ s^–1^ during the day), relative humidity of 70% during the day and 65% at night. The branches were kept in the growth chamber for 42 days (6 weeks). The forcing duration of 42 days corresponded to the maximum period during which the branches could be kept healthy under the abovementioned conditions (no severe tissue decomposition under water and no visible desiccation of the upper parts of the branches). The water in the buckets was changed once a week. The bases of the branches were not cut while kept in the growth chamber.

### Budbreak percentages after artificial forcing and time to budbreak

Every second day, all buds were examined for budbreak, which was defined as stage 53 of the BBCH scale (German: Biologische Bundesanstalt, Bundessortenamt und CHemische Industrie) for apple (Meier 2018). At stage BBCH 53, the protective scales that cover the buds begin to open up, revealing whether the bud contains flower parts inside. The time to budbreak (i.e., the number of days under forcing conditions that the buds needed to reach the stage of budbreak) and bud type (floral or vegetative) were documented for each bud that reached the stage of budbreak. Budbreak percentages were calculated as the share of all buds of a particular type (spur, terminal and lateral, and also terminal + spur buds) that showed budbreak.

In order to compare the budbreak percentages of the attached branches in the orchard and of the detached branches under budbreak forcing conditions, six branches from each of the studied apple cultivars (one branch per tree) were sampled at the stage of paradormancy (on 30 June 2021). The branches were defoliated and kept under budbreak forcing conditions as described above. In the orchard, six apple branches of ‘Nicoter’ and six apple branches of ‘Topaz’ (one branch per tree) were labeled and defoliated. The numbers of terminal and spur buds that reached the stage of budbreak were counted after 42 days. Each branch served as a replicate. The budbreak percentages were calculated as the share of all buds of a particular type (for terminal and spur buds separately) that showed budbreak.

### Water content in apple spur buds

We collected 20 spur buds (five buds in four replicates) per cultivar at weekly intervals and analysed them for their water content (WC). Immediately after sampling, brown bud scales were removed, the buds were placed in safe-lock tubes (2-mL), weighed on an analytical balance (Satorius TE64, Göttingen, Germany) and snap-frozen in liquid nitrogen. The samples were stored at −30 °C until freeze-dried. All the steps of freeze-drying were completed within 13 days (Freeze-dryer of Dieter Piatkowski, Munich, Germany). The temperature of the drying cabinet was set to −80 °C, whereas the initial sample holder plate temperature was −30 °C. The sample holder plate temperature was increased by 5 °C every day, reaching 30 °C on Day 13. Fresh weight (FW) and DW of spur buds were determined using the same analytical balance (Satorius TE64, Göttingen, Germany). The WC in buds was calculated as % of FW.

### Tabuenca’s test

Tabuenca’s test (Tabuenca 1964) was applied for ‘Nicoter’ only (experimental season of 2021 to 2022). We used the procedures that were described by El Yaacoubi et al. (2016) for apple, with some modifications. Specifically, 15 newly formed long shoots (~40 cm in length) were cut from ‘Nicoter’ trees at weekly intervals from 26 November 2021 until 16 February 2022. These shoots were placed in water and kept in the growth chamber (see the conditions above) for 7 days. At the end of this period, terminal buds were cut, weighed on the analytical balance and freeze-dried (see the conditions above) to determine DW and WC of the buds. Terminal buds from another 15 shoots of ‘Nicoter’, which were collected in the orchard on the same dates, were immediately weighed on the analytical balance, freeze-dried and used as control. At the time when the terminal buds were cut from the shoots, the brown bud scales were completely removed from each bud. The WC in buds (calculated as % of FW) was not a part of the original Tabuenca’s test (Tabuenca 1964).

### Shoot growth cessation and leaf fall

Shoot growth cessation and leaf fall were documented in the growing season of 2021. In order to determine the timing of shoot growth cessation, 25 randomly selected shoots from five trees of ‘Nicoter’ and ‘Topaz’ (five shoots per tree) were labeled and measured at weekly intervals from 17 May until 13 July and also on 27 July and on 26 August. The beginning of shoot growth cessation was defined as the time when the dynamics of shoot length increment slowed down and hence deviated from their linear development (Fig. S1 available as Supplementary data at *Tree Physiology* Online). Leaf fall for both ‘Nicoter’ and ‘Topaz’ in 2021 began on 4 November and continued until 1 December. As the beginning of leaf fall, we visually estimated the date when ~10% of leaves had fallen from the trees.

### Dates of full bloom and budbreak in the orchard

Full bloom (75% of flowers open, determined by visual estimation) of ‘Nicoter’ and ‘Topaz’ occurred on 25 April in 2019 and on 30 April in 2021, respectively. For the interpretation of some results of the experiment conducted in 2021 to 2022, we use the date of natural budbreak in the orchard. Natural budbreak in the orchard for both ‘Nicoter’ and ‘Topaz’ began on 20 March 2022. In order to ensure data comparability with other studies, we also specify dates in terms of days after full bloom (DAFB), in particular in our illustrations and Supplementary materials.

### Calculation of chilling and forcing

Chilling accumulation was calculated using three chill models: the Utah Chill Model for apple, with Utah Chill Units as an output (Shaltout and Unrath 1983), the Chill Hours Model (with Chill Hours being hours with temperatures between 0.0 °C and 7.2 °C), and the Dynamic Model which quantifies chill in Chill Portions (Fishman et al. 1987*a*, 1987*b*). Chill accumulation was calculated starting from the month when the models detected the first chill units/hours/portions. For the experimental orchard at the University of Hohenheim, the chilling period began in September (the exact numbers of chill hours, units or portions accumulated from 1 September of each year and until each sampling date are specified in Table S1 available as Supplementary data at *Tree Physiology* Online). Forcing accumulation was calculated using the Growing Degree Hours model with equations provided by Luedeling et al. (2009). Hourly temperature data were obtained from the weather station at Hohenheim, Germany, which was located 200 m away from the experimental apple trees.

### Statistical tests and illustrations

Statistical data analyses included Student’s t-test (with a significance level of *P* < 0.05) and the Pearson correlation test, which were applied using SigmaPlot 14.0 (Systat Software GmbH). All figures that are shown in the current work were created using the same software.

## Results

### Budbreak percentages

**Figure 1:**
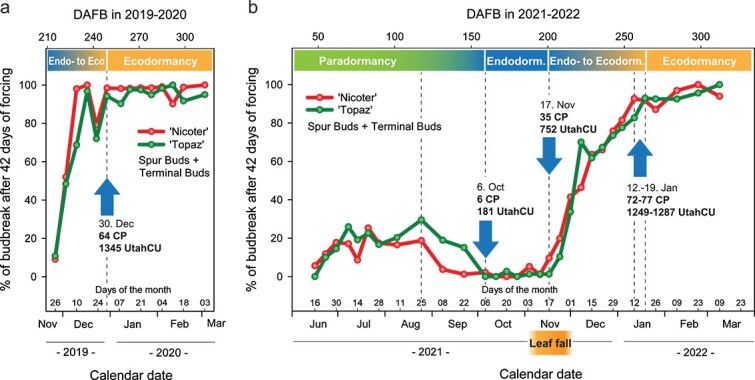
Budbreak percentages of terminal and spur buds (calculated together) on apple branches sampled from ‘Nicoter’ and ‘Topaz’ trees in 2019 to 2020 (a) and 2021 to 2022 (b). The branches were kept under budbreak forcing conditions for 42 days. The x-axis shows Tuesdays (a) and Wednesdays (b), since the sampling was primarily done on Tuesdays in 2019 to 2020 and on Wednesdays in 2021 to 2022. For convenient comparison of calendar dates with the exact number of days after full bloom (DAFB), the reader is referred to Table S1 available as Supplementary data at *Tree Physiology* Online. CP—Chill Portions, UtahCU—Utah Chill Units.

In the experiment that was conducted in 2019 to 2020, the budbreak percentages of spur and terminal buds (calculated together) for both ‘Nicoter’ and ‘Topaz’ were relatively low at the beginning of the sampling period (26 November 2019), at 9.1% for ‘Nicoter’ and 10.8% for ‘Topaz’. Over the following 3 to 4 weeks, the budbreak percentages increased rapidly, exceeding 90% and stabilizing at this level starting from 30 December for ‘Nicoter’ and ‘Topaz’ (Fig. 1a). The time span of this experiment, however, did not allow the calculation of budbreak percentages in early autumn (at the beginning of the chilling period). Such data would provide a more complete picture of the budbreak behavior during the onset of endodormancy. For this reason, the sampling period was extended considerably in 2021 to 2022. The new sampling period began in mid-June (the period of active bud formation for the following year in the northern hemisphere) and continued until 9 March, or 11 days prior to natural budbreak in the orchard. The extended sampling period allowed observing the changes in budbreak behavior during the whole developmental cycle of apple buds and to thereby distinguish the three dormancy phases (para-, endo- and ecodormancy), as well as the transition periods between them (Fig. 1b).

During paradormancy, budbreak forcing conditions combined with defoliation of apple branches could to some extent stimulate budbreak of spur buds, whereas terminal buds remained inactive, showing no visible changes even after 42 days of forcing in the growth chamber (Fig. S2a-d available as Supplementary data at *Tree Physiology* Online). All the spur buds that resumed their growth in June and July under forcing conditions were exclusively vegetative buds. The first budbreak of flower buds was observed on 9 August 2021 for ‘Nicoter’ and on 25 August 2021 for ‘Topaz’ (Fig. S3 available as Supplementary data at *Tree Physiology* Online).

The establishment of endodormancy, as determined by budbreak percentages, occurred at the end of September 2021 for ‘Nicoter’ and at the beginning of October 2021 for ‘Topaz’. During the transition from para- to endodormancy, the weekly mean temperature in the orchard decreased from 16.4 °C to 12.2 °C (Fig. S4; Table S2 available as Supplementary data at *Tree Physiology* Online). After the establishment of endodormancy, budbreak percentages of both apple cultivars were close to zero. In mid-November of 2021, budbreak percentages started to increase, indicating the beginning of the transition from endo- to ecodormancy. Budbreak percentages of ‘Nicoter’ and ‘Topaz’ exceeded 90% in mid-January 2022 (Fig. 1b). Such high budbreak percentages were thus achieved ~2 to 3 weeks later in the winter of 2021 to 2022 compared with 2019 to 2020.

According to the dynamic model for chill accumulation, endodormancy release in the studied apple cultivars occurred when 64 Chill Portions (CP) had accumulated in the experimental season of 2019 to 2020. In contrast, in the experimental season of 2021 to 2022, the trees had been exposed to 72 to 77 CP prior to endodormancy release (Fig. 1a and b; Table S1 available as Supplementary data at *Tree Physiology* Online). The Utah Model showed accumulation of 1345 Utah Chill Units (UtahCU) in the experimental season of 2019 to 2020 and of 1249 to 1287 UtahCU in the experimental season of 2021 to 2022 by the time when endodormancy release occurred for ‘Nicoter’ and ‘Topaz’ (Fig. 1a and b; Table S1 available as Supplementary data at *Tree Physiology* Online).

The temporal progression of budbreak percentages during the dormancy period differed between spur, terminal and lateral buds. Our data showed that among all bud types of ‘Topaz’, terminal buds were the first to reach a high budbreak percentage (>90%), followed by spur buds of the same cultivar. In contrast, terminal and spur buds of ‘Nicoter’ crossed the 90% threshold relatively simultaneously (Fig. S5b and c available as Supplementary data at *Tree Physiology* Online). The lateral buds of both apple cultivars had much lower budbreak percentages even 11 days prior to natural budbreak in the orchard (9 March 2022): 65.9% and 48.9% for ‘Nicoter’ and ‘Topaz’, respectively (Fig. S5d available as Supplementary data at *Tree Physiology* Online).

### Timing and synchrony of budbreak

**Figure 2:**
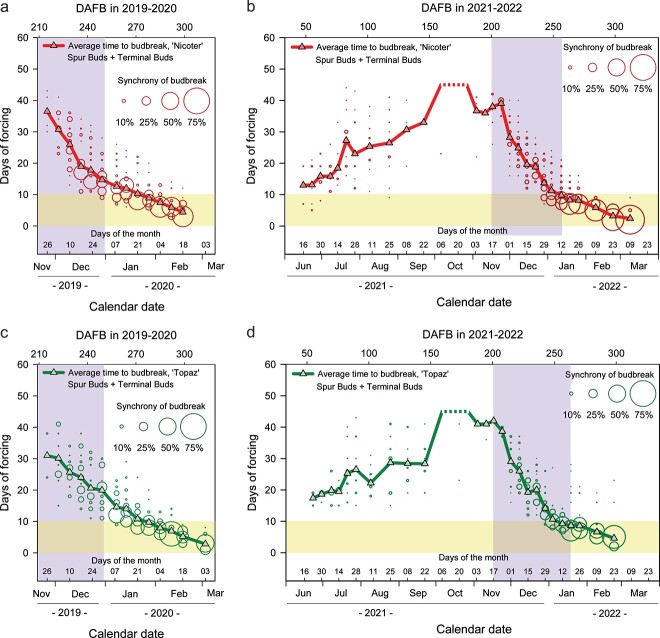
Mean time to budbreak (line plot) and budbreak synchrony (bubble plot) for ‘Nicoter’ (a, b) and ‘Topaz’ (c, d) in 2019 to 2020 (a, c) and in 2021 to 2022 (b, d). The branches were kept under budbreak forcing conditions for 42 days. The size of each ‘bubble’ indicates the percentage of buds that reached the stage of budbreak among the total number of buds (spur + terminal) on the corresponding day of forcing. The total number of buds was obtained from all the branches (each cultivar) that were sampled at the same date (plotted on x-axis). Broken lines show either the complete absence of budbreak at the corresponding dates or poor budbreak (not enough data for the statistical analysis). The purple area (vertical bar) indicates the transition phase from endo- to ecodormancy (determined according to the data shown in Fig. 1a and b. The yellow area (horizontal bar) marks the period of 10 days of forcing that is widely used to determine chilling requirements of fruit trees.

The time that apple buds require to reach the stage of budbreak under forcing conditions may be useful as an indicator of dormancy depth. In 2021 to 2022, the mean time to budbreak for the newly formed spur buds of both apple cultivars increased during the vegetative period (June–August; Fig. 2b and d; Fig. S2a and b available as Supplementary data at *Tree Physiology* Online), whereas terminal buds did not show any budbreak (Fig. S2c and d available as Supplementary data at *Tree Physiology* Online).

At the establishment of endodormancy (early October), the budbreak percentages for spur buds were close to zero and hence the mean time to budbreak could not be calculated. During the transition phase from endo- to ecodormancy, the mean time to budbreak (for terminal + spur buds) decreased from 38.0 to 9.8 days for ‘Nicoter’ and from 42.0 to 8.7 days for ‘Topaz’. Besides, the synchrony of budbreak considerably increased towards the time of natural budbreak in the orchard. In particular, by the end of the transition from endo- to ecodormancy (determined by the budbreak percentages), > 90% of terminal and spur buds reached the stage of budbreak between 7 and 21 days of forcing for both apple cultivars (Fig. 2b and d). This interval became much shorter 3 weeks prior to natural budbreak in the orchard (23 February 2022), with > 90% of buds breaking after 2 to 5 days of forcing for both ‘Nicoter’ and ‘Topaz’.

A different picture of budbreak synchrony was observed in 2019 to 2020, when endodormancy release occurred earlier than in 2021 to 2022. By the time of endodormancy release, budbreak was less synchronized in 2019 to 2020 compared with 2021 to 2022 (Fig. 2a and c). The synchrony of budbreak, however, was markedly improved prior to natural budbreak in the orchard.

### Water content in apple spur buds and bud weight

**Figure 3:**
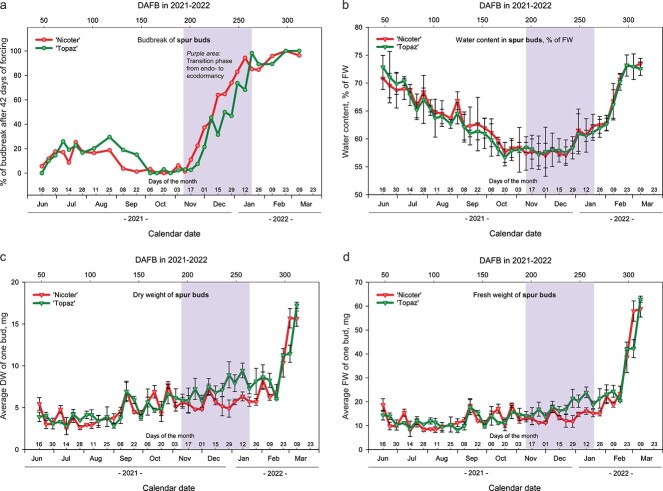
Budbreak percentages of spur buds of ‘Nicoter’ and ‘Topaz’ after 42 days of forcing (a); characteristics of spur buds collected in the orchard (no artificial forcing applied): WC (b), DW (c) and FW (d) in 2021 to 2022. The purple area (vertical bar) indicates the transition phase from endo- to ecodormancy (determined according to the data shown in Fig. 1b). Error bars indicate standard deviation.

During the sampling period of 2021 to 2022, we determined the temporal progression of average fresh and dry weight of spur buds in the orchard (FW and DW, respectively; Fig. 3c and d). The average DW of spur buds increased very slowly from June 2021 until February 2022, before rising sharply between 16 February and 9 March 2022 (Fig. 3c). This rapid increase of bud weight in the orchard started about 12 weeks after the detectable rise of budbreak percentages under 42 days of forcing (24 November 2021; Fig. 3a) or about 4 weeks prior to natural budbreak in the orchard for both ‘Nicoter’ and ‘Topaz’. At the beginning of the experimental period (16 June 2021), the average DW of spur buds was 5.4 mg for ‘Nicoter’ and 3.9 mg for ‘Topaz’ whilst 11 days prior to natural budbreak in the orchard (9 March 2021) the DW of spur buds reached 15.6 mg for ‘Nicoter’ and 17.2 mg for ‘Topaz’. The average FW of the same spur buds showed a very similar trend to that of DW (Fig. 3d).

During the period of bud development (June–August 2021), the WC of spur buds in the orchard exhibited considerable changes over time but did not differ significantly between the apple cultivars. On 16 June 2021, the WC of spur buds was 68.9% to 75.0% depending on the cultivar (Fig. 3b). It gradually declined during the vegetative period until reaching its minimum level on 20 October 2021 (55.2% to 59.4%) when the trees were in the phase of endodormancy (Fig. 3a). At the end of December 2021, the WC in spur buds started increasing again, reaching levels that were comparable to those when the buds were newly formed (Fig. 3b).

Pearson correlation between budbreak percentages of spur buds and other spur bud parameters, such as FW, DW and WC (whole sampling period, 31 sampling dates, 16 June 2021–9 March 2022) showed no significant relationship between budbreak percentages and WC for both ‘Nicoter’ and ‘Topaz’ (Fig. S6a and b available as Supplementary data at *Tree Physiology* Online). The correlation coefficients for budbreak percentages vs DW were 0.49 for ‘Nicoter’ and 0.63 for ‘Topaz’ whereas the correlation between budbreak percentages vs FW was 0.50 and 0.65 for ‘Nicoter’ and ‘Topaz’, respectively (Fig. S6c–f available as Supplementary data at *Tree Physiology* Online). Considering only the transition period from endo- to ecodormancy (11 sampling dates, 11 November 2021–19 January 2022), the correlation coefficients for budbreak percentages vs WC were improved, however, this sample size was critically low for any correlation tests (data not shown).

The correlation tests for the mean time to budbreak vs FW, DW and WC showed negative relationships between all tested parameters with correlation coefficients ranging between −0.31 and −0.65 depending on tested parameter pairs and cultivars (Fig. S7a–f available as Supplementary data at *Tree Physiology* Online).

### Tabuenca’s test

**Figure 4:**
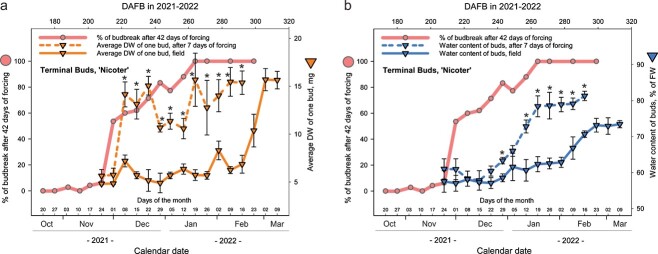
Tabuenca’s test with terminal buds of ‘Nicoter’ (a) and WC in terminal buds of ‘Nicoter’ (b), measured directly after sampling in the orchard (solid line with triangle symbols) and after 7 days of forcing (dashed line with triangle symbols). Error bars indicate standard deviation. Asterisks mark significant differences between DW of buds with/without forcing (a) and between WC of buds with/without forcing (b) at *P* < 0.05.

Tabuenca’s test is based on comparison of the DWs of terminal buds after two contrasting treatments: 7 days of forcing and without forcing. The first detectable increase of bud DW after 7 days of forcing is expected to indicate the timing of endodormancy release. Tabuenca’s test showed that the DW of ‘Nicoter’ terminal buds that were exposed to forcing increased on 8 December 2021, in contrast to the buds without forcing (Fig. 4a). This increase of DW coincided with the time when the budbreak percentages of ‘Nicoter’ terminal buds reached 60% after 42 days of forcing (Fig. 4a; Table S1 available as Supplementary data at *Tree Physiology* Online). While conducting Tabuenca’s test, we also measured the WC of terminal buds in both treatments. Clear differences in WCs between the buds under field and growth chamber conditions were detectable from 12 January 2021. One week after this date, budbreak percentages of ‘Nicoter’ terminal buds first exceeded 90% after a 42-day forcing period (Fig. 4b).

## Discussion

Over the past few decades, numerous studies have addressed various questions related to fruit tree dormancy (Campoy et al. 2011*a*; Fadón et al. 2020). However, there is hardly any other topic in horticulture with so many speculations and assumptions that are not supported by robust experimental evidence. One of the major limiting factors for dormancy-related experiments is the limited availability of high-tech growth chambers, which are important for elucidating the role of chilling and forcing temperatures in dormancy release and hence in determining the timing of budbreak. Moreover, the heterogeneity of growth chambers and heated greenhouses used for dormancy experiments has given rise to a host of experimental methods, the results of which are hardly comparable. For example, in many works a common threshold of 50% budbreak is used to determine chilling requirements (Ghariani and Stebbins 1994; Measham et al. 2017; Wenden et al. 2018; Parkes et al. 2020). However, other experimental conditions are prone to considerable methodological variations. Specifically, Parkes et al. (2020) applied 14 days of forcing at 25 °C and continuous light for apple shoots, Ghariani and Stebbins (1994) used 28 days of forcing for pear shoots at 24/19 °C (day/night), whereas Wenden et al. (2018) determined chilling requirements of sweet cherry by exposure of branches to 10 days of forcing at 25 °C and 16 h of daily lighting. Other parameters, such as air humidity, light intensity or weekly cutting of the bases of shoots or branches, may also influence the results.

The threshold of 50% budbreak that is often used to determine chilling requirements in fruit trees originated from early experiments by Weinberger (1950), who used 1-year-old shoots to determine dormancy release in peach. Weinberger clearly declared that the parameters used for the estimation of chilling requirements for peach (a threshold of 50% budbreak after 3 weeks in warmth; the temperature was not specified) were arbitrary. Recent experiments with ‘Fuji’ and ‘Royal Gala’ apple trees, which received a natural dose of chill in the orchard, demonstrated that budbreak percentages of terminal and spur buds varied between 88% and 100%, depending on the cultivar, whereas the budbreak percentages for lateral buds were lower and ranged between 57% and 72% (Koehler and Milyaev 2022). This indicates that the threshold of 50% budbreak, if applied irrespective of cultivar or bud type, might deliver misleading conclusions regarding the chilling requirements of certain tree species, including apple. The results of the current work confirmed that lateral buds of apple shoots showed relatively low budbreak percentages even at the stage of ecodormancy: 65.9% for ‘Nicoter’ and 48.9% for ‘Topaz’ (Fig. S5d and Table S1 available as Supplementary data at *Tree Physiology* Online).

A completely different picture was observed for terminal and spur buds, which were predominantly flower buds in ‘Nicoter’ and ‘Topaz’ (Fig. S3a, b and Fig. S8a, b available as Supplementary data at *Tree Physiology* Online) and are therefore shown together in Fig. 1a and b. The budbreak percentages of terminal and spur buds reached > 90%, as was shown for both experimental seasons, in 2019 to 2020 and 2021 to 2022. Our data suggest that the threshold of budbreak percentage that indicates the fulfillment of chilling requirements cannot be universal for every tree species, cultivar and bud type (terminal, spur, lateral). For experiments that aim to study chilling requirements of trees, it is important to check the maximum budbreak potential of the trees that have been exposed to sufficient chill. To determine chilling requirements, the threshold of budbreak percentage should therefore be set close to the maximum budbreak potential that represents healthy budbreak of the studied trees in spring. This budbreak potential needs to be determined in a given experimental year and for all genotypes and bud types that are involved in the study.

The current work aimed to demonstrate the dormancy progression in apple by exposure of detached apple branches to artificial forcing in a growth chamber. The data illustrated that budbreak percentages, the mean time to budbreak and synchrony of budbreak varied throughout the dormancy period. Our results showed that budbreak percentages of both terminal and spur buds of ‘Nicoter’ and ‘Topaz’ shaped a trendline that clearly marked the three known dormancy phases, i.e. para-, endo- and ecodormancy (Fig. 1a and b). This trendline suggests a population effect where some buds in the population reach endodormancy release earlier and some later. Endodormancy release was defined as the time when > 90% of terminal and spur buds showed budbreak within 42 days of forcing and further maintained this tendency. However, the synchrony of budbreak at the time of the defined endodormancy release differed considerably between the two experimental periods. In 2019 to 2020, when endodormancy release occurred earlier, budbreak was more asynchronous compared with 2021 to 2022 (Fig. 2a–d). Therefore, if dormancy release is only determined by the budbreak percentages, optimal synchrony of budbreak might still not be achieved. Asynchronous budbreak could lead to uneven fruit set and ripening and hence to an extended picking time, which is undesirable for commercial growers (Roger et al. 1982). Our data showed that the fewer days of forcing were required to achieve > 90% of budbreak, the better was budbreak synchrony (Fig. 2a–d). In the current study with the branches of ‘Nicoter’ and ‘Topaz’, budbreak was relatively synchronous when > 90% of buds reached the stage of budbreak within 10 days of forcing. It seems likely that apple trees grown in temperate climates use the period of ecodormancy with low forcing temperatures in spring to synchronize budbreak.

The question of whether the timing or the synchrony of budbreak needs to be considered for objective determination of dormancy release in fruit trees remains obscure. On one hand, to capture the fraction of the ecodormancy period that is characterized by synchronous budbreak in combination with high budbreak percentages in apple, applying only 15 to 20 days of forcing may be sufficient. On the other hand, shortening the forcing period (e.g. from 42 to 20 days) in such experiments will inevitably lead to the loss of valuable data on the progression of the mean time to budbreak and of budbreak percentages and thus on their response to the natural combination of chilling and forcing temperatures in the orchard. Such high-resolution data would be particularly interesting for the precise modeling of plant responses to ambient temperature as well as for the elucidation of the molecular and genetic background of dormancy.

Besides conducting the growth chamber experiment described in the current work, we tested such parameters as bud weight and WC in buds as well as Tabuenca’s test, which were described in previous works as potential methods to determine the bud dormancy status (Brown and Kotob 1957; Tabuenca 1964; Malagi et al. 2015; Kaufmann and Blanke 2017). Our results indicated that neither WC in buds nor average bud weight (both DW and FW) showed any similarities to the patterns of budbreak percentages in ‘Nicoter’ and ‘Topaz’ (Fig. 3a–d). This was confirmed by the correlation tests that were carried out with WC, DW and FW vs budbreak percentages, which also emphasized cultivar differences (Fig. S6a–f available as Supplementary data at *Tree Physiology* Online). The clear increases of both WC in buds and bud weight in the orchard were detectable much later than the increase of budbreak percentages after 42 days of forcing. Therefore, we concluded that these parameters cannot replace time-consuming growth chamber experiments, which are important for distinguishing dormancy phases, identifying their transition periods and determining chilling requirements in apple.

In our experiment, Tabuenca’s test indicated neither the beginning of the transition phase from endodormancy to ecodormancy, nor endodormancy release. However, the test was able to indicate the date when over a half of terminal buds (60%) reached the stage of budbreak after 42 days of forcing. Moreover, Tabuenca’s test detected a marked increase of both DW and WC of buds after 7 days of forcing, when the trees were at the transition phase from endo- to ecodormancy. This suggests that Tabuenca’s test has potential to be used to identify endodormancy release in apple after some methodological adjustments (e.g. duration of forcing) and further validation. This could be accomplished through multi-year studies, in which different cultivars are exposed to artificial forcing. In such studies, besides applying Tabuenca’s test, the dynamics of budbreak percentages (by frequent sampling and subsequent artificial forcing) should also be determined.

Previous studies have indicated that growth cessation and bud dormancy may be driven by the same environmental (Heide and Prestrud 2005) and genetic (Moser et al. 2020) factors. In the current study, the dynamics of shoot growth were documented in the experimental period of 2021 to 2022 to identify connections between shoot growth cessation and budbreak percentages after forcing. Shoot growth cessation was detected at the end of June (2021) for ‘Nicoter’ and at the beginning of July (2021) for ‘Topaz’ (Fig. S1 available as Supplementary data at *Tree Physiology* Online). During nearly 55 days after these events, we detected no changes in the budbreak behavior of the studied cultivars under forcing conditions (Fig. 1b). Therefore, we concluded that shoot growth cessation may not have a direct connection to the onset of dormancy in apple.

Leaf fall has been widely discussed as possibly being linked with the onset of endodormancy (Saure 1985). In our experiment, leaf fall was documented only in the experimental season of 2021 to 2022. The data showed that the period of leaf fall in autumn 2021 coincided with the beginning of the transition phase from endo- to ecodormancy (Fig. 1b). Possible biosynthesis of dormancy-inducing factors in leaves has been hypothesized in previous studies (e.g., Lang et al. 1987). This notion is supported by defoliation apparently stimulating budbreak, as has been discussed by Campoy et al. (2011*a*) and was also shown in the present work.

Comparison of budbreak percentages of defoliated apple branches in the growth chamber (detached branches) and in the orchard (attached branches) showed similar budbreak behavior under both conditions. Specifically, defoliation of apple branches on 30 June 2021 was unable to induce budbreak of terminal buds, whereas, depending on the cultivar, 7.5% to 8.2% and 20.8% to 25.2% of spur buds reached the stage of budbreak within 42 days in the orchard and in the growth chamber, respectively. These differences, however, were not statistically significant (Fig. S9a and b available as Supplementary data at *Tree Physiology* Online). Future research should test whether the leaves export certain mobile compounds to the adjacent buds to prevent premature budbreak during the vegetative period, whether leaf fall in temperate climates marks the starting point for buds to effectively perceive chilling temperatures for dormancy release, and whether the synchrony of leaf fall dictates the synchrony of endodormancy release in buds.

In summary, growth chamber experiments with budbreak forcing conditions provided new insights on dormancy progression in apple. Budbreak percentages and the mean time to budbreak calculated for each of the 14 sampling weeks in 2019 to 2020 and for each of the 31 sampling weeks in 2021 to 2022 illustrated dormancy dynamics in ‘Nicoter’ and ‘Topaz’. Moreover, the budbreak percentages of ‘Nicoter’ and ‘Topaz’ marked the three known dormancy stages: para-, endo- and ecodormancy, as well as transition periods between them. The mean time to budbreak and synchrony of budbreak varied markedly over the experimental periods, indicating that these parameters may be considered for better understanding of plant responses to ambient temperature during dormancy. The increase of both WC in buds and bud weight in the orchard were only detectable several weeks after a clear rise of budbreak percentages under forcing conditions for both apple cultivars. This indicated that neither WC in buds nor DW or FW of buds collected in the orchard were suitable for determining chilling requirements and hence endodormancy release in ‘Nicoter’ and ‘Topaz’. However, the conditions of Tabuenca’s test stimulated a significant increase of WC and DW of terminal buds in ‘Nicoter’ during the transition period from endo- to ecodormancy. This makes Tabuenca’s test promising for the identification of endodormancy release in apple after its adjustments, validation and further trials investigating the degree of cultivar specificity.

The dormancy mechanism in fruit trees appears to be a complex system, in which many unknown constituents are still to be revealed. To unravel this mechanism, long-term experiments with fruit trees using budbreak forcing conditions and standardized methods are required. Such experiments are necessary to understand how trees react to the specific winter conditions that occur in a particular year. Comparison of budbreak behavior over several years is essential for understanding how trees ‘count’ chilling and forcing temperatures in order to complete their period of endodormancy by the beginning of the next vegetative period and to synchronize budbreak in spring.

## Supplementary Material

Suppl_Fig_S1_tpae112

Suppl_Fig_S2_tpae112

Suppl_Fig_S3_tpae112

Suppl_Fig_S4_tpae112

Suppl_Fig_S5_tpae112

Suppl_Fig_S6_tpae112

Suppl_Fig_S7_tpae112

Suppl_Fig_S8_tpae112

Suppl_Fig_S9_tpae112

Suppl_table_1_tpae112

Suppl_table_2_tpae112

## Data Availability

The data that support the findings of this study are available in supplementary materials. Specific data that were not included in the supplementary materials are available on request from the corresponding author, A.M.
