# Supplementary material for: Identifying indicators of apple bud dormancy status by exposure to artificial forcing conditions
Source: Tree Physiol. 2024 Aug 31;44(10):tpae112. doi: 10.1093/treephys/tpae112 (PMC11447376; doi:10.1093/treephys/tpae112)
Supplement: Suppl_Fig_S1_tpae112 [file suppl_fig_s1_tpae112.pdf]

Identifying indicators of apple bud dormancy status by exposure to artificial forcing conditions

Anton Milyaev[1,2], Ute Born[2], Elke Sprich[2], Michael Hagemann[2], Henryk Flachowsky[3] and Eike Luedeling[1]

[1]University of Bonn, Horticultural Sciences, Institute of Crop Science and Resource Conservation (INRES), Auf dem Hügel 6, 53121, Bonn, Germany

[2]University of Hohenheim, Institute of Crop Science, Section Production Systems of Specialty Crops (340f), Emil-Wolff-Street 25, 70599 Stuttgart, Germany

[3]Julius Kühn Institut (JKI) - Institute for Breeding Research on Fruit Crops, Federal Research Centre for Cultivated Plants, Pillnitzer Platz 3a, 01326 Dresden, Germany

Supplementary figures

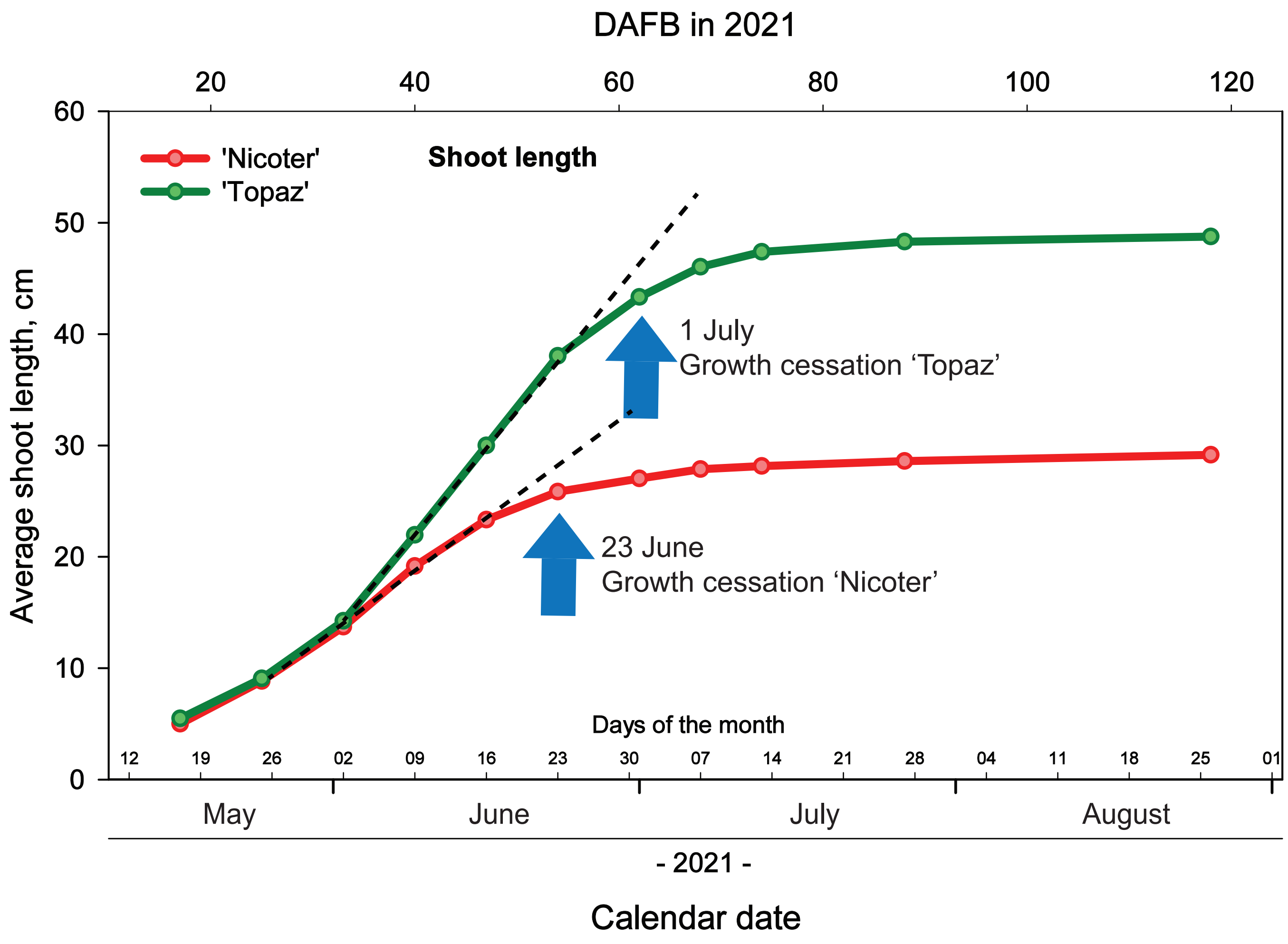

Suppl. Figure 1. Dynamics of shoot growth for 'Nicoter' and 'Topaz' in summer 2021. Each data point is the average of 25 shoots that were measured during the growing season. The time of shoot growth cessation is marked with blue arrows.
