# Supplementary material for: Identifying indicators of apple bud dormancy status by exposure to artificial forcing conditions
Source: Tree Physiol. 2024 Aug 31;44(10):tpae112. doi: 10.1093/treephys/tpae112 (PMC11447376; doi:10.1093/treephys/tpae112)
Supplement: Suppl_Fig_S2_tpae112 [file suppl_fig_s2_tpae112.pdf]

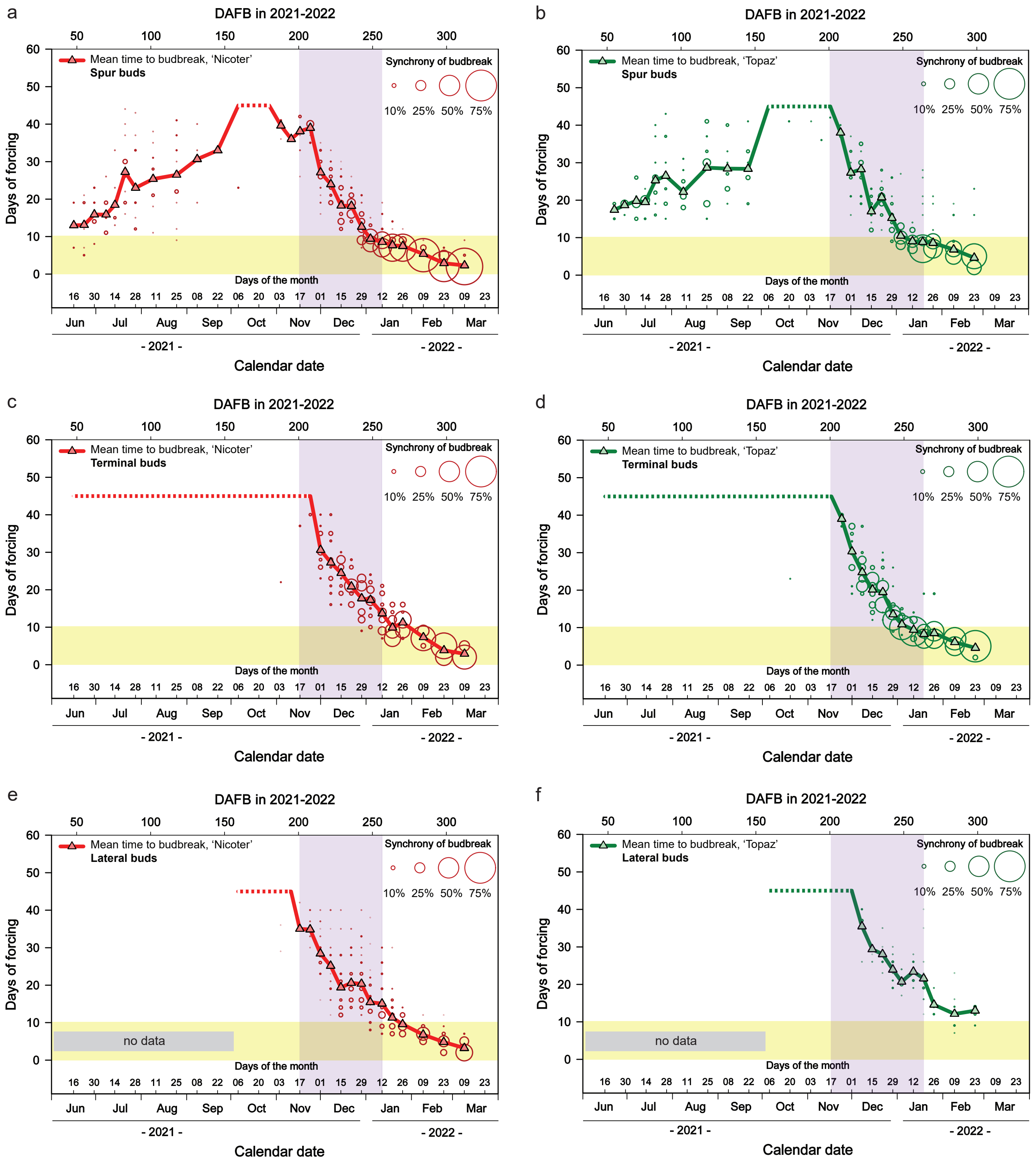

Suppl. Figure 2. Mean time to budbreak (line plot) and budbreak synchrony (bubble plot) for spur (a, b), terminal (c, d) and lateral buds (e, f) of 'Nicoter' (a, c, e) and 'Topaz' (b, d, f) in 2021-2022.

The branches were kept under budbreak forcing conditions for 42 days. The size of each "bubble" indicates the percentage of buds that reached the stage of budbreak as a fraction of the total number of buds (spur, terminal or lateral, separately) on the corresponding day of forcing. The total number of buds was obtained from all the branches (each cultivar) that were sampled on the same date (plotted on x-axis). Broken lines show either the complete absence of budbreak on the corresponding dates or poor budbreak (not enough data for statistical analysis). The purple area indicates the transition phase from endo- to ecodormancy (determined according to the data shown in Figure 1). The yellow area marks the period of 10 days of forcing that is widely used to determine chilling requirements of fruit trees.
