# Supplementary material for: Identifying indicators of apple bud dormancy status by exposure to artificial forcing conditions
Source: Tree Physiol. 2024 Aug 31;44(10):tpae112. doi: 10.1093/treephys/tpae112 (PMC11447376; doi:10.1093/treephys/tpae112)
Supplement: Suppl_Fig_S3_tpae112 [file suppl_fig_s3_tpae112.pdf]

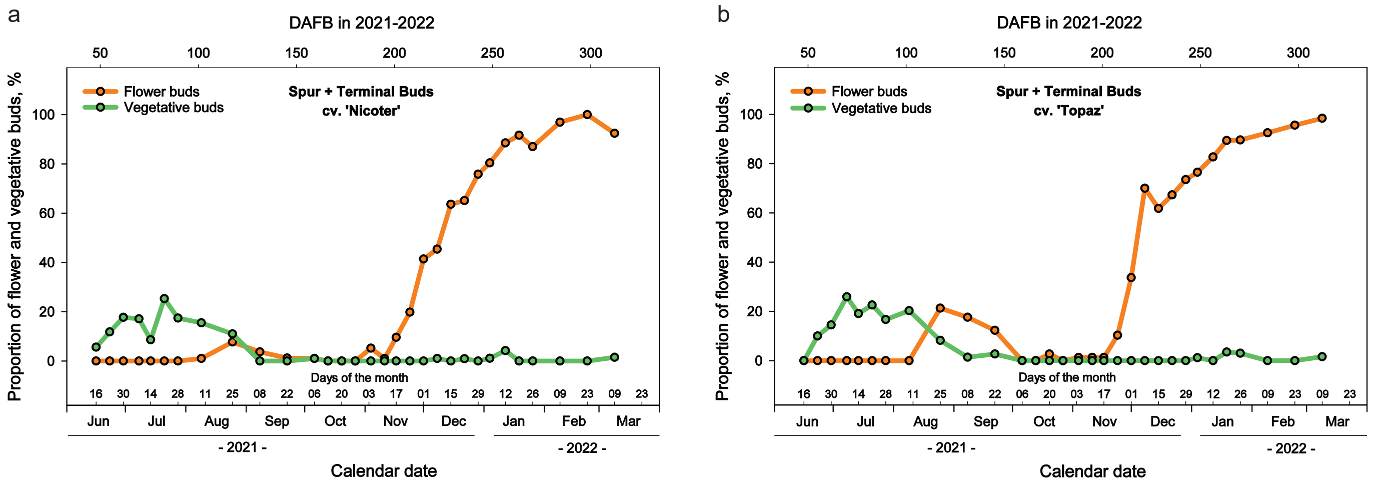

Suppl. Figure 3. Budbreak percentages of terminal and spur buds (calculated together) on apple branches that were sampled from ‘Nicoter’ (a) and ‘Topaz’ (b) trees grown in the orchard in 2021-2022.

The branches were kept under budbreak forcing conditions for 42 days. The Figure shows the proportion of vegetative (green line) and flower buds (orange line) to the total number of buds that reached the stage of budbreak (also shown in Figure 1b in the main text of the manuscript). Example: If the green line reaches 20% and the orange line indicates 10% on a particular calendar date, this means that among the total number of terminal and spur buds (calculated together), 30% of buds reached the stage of bud-break. Out of these, 2/3 of the buds were vegetative whereas 1/3 were flower buds.
