# Supplementary material for: Identifying indicators of apple bud dormancy status by exposure to artificial forcing conditions
Source: Tree Physiol. 2024 Aug 31;44(10):tpae112. doi: 10.1093/treephys/tpae112 (PMC11447376; doi:10.1093/treephys/tpae112)
Supplement: Suppl_Fig_S4_tpae112 [file suppl_fig_s4_tpae112.pdf]

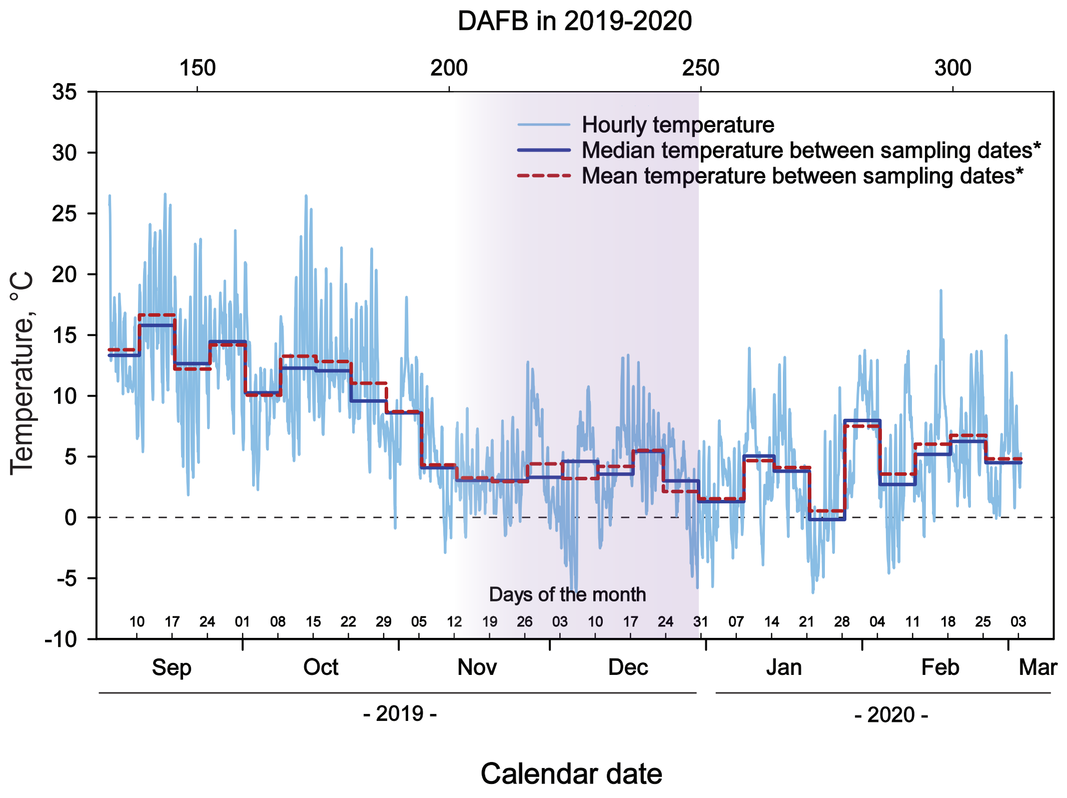

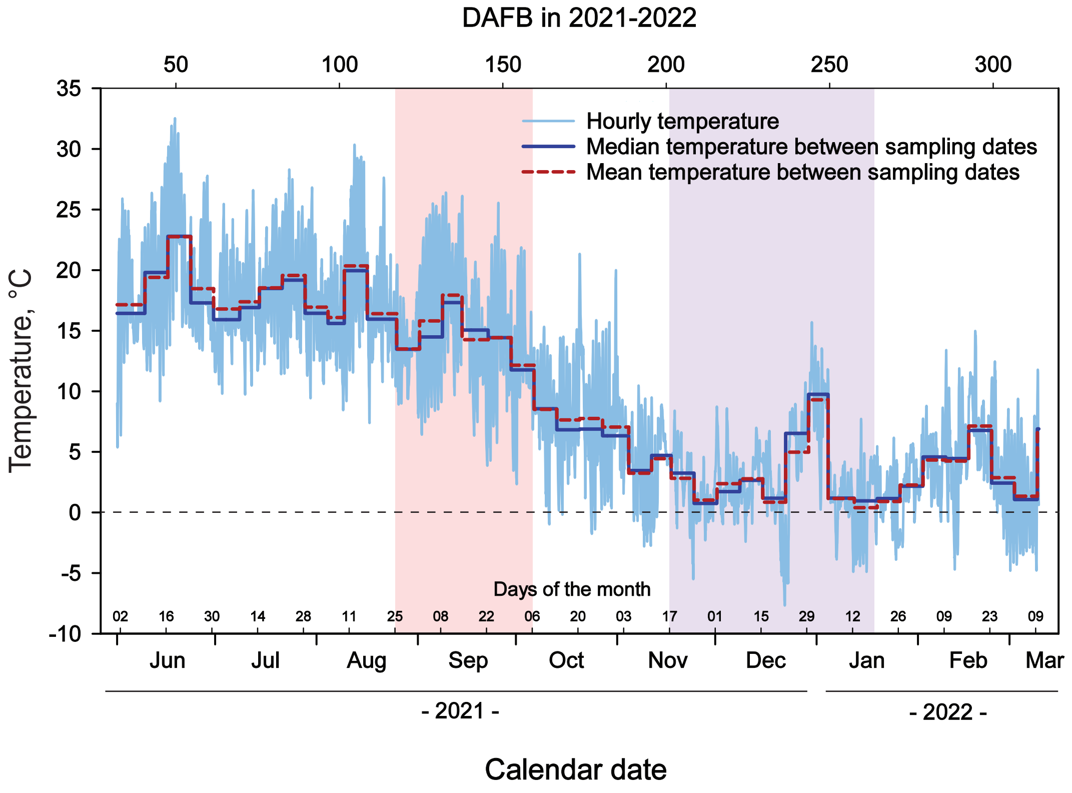

Suppl. Figure 4. Temperature conditions in the orchard during the experimental periods of 2019-2020 (top figure) and 2021-2022 (bottom figure).

The values for mean and median temperatures can be found in Suppl. Table 2. The orange area shows the transition phase from para- to endodormancy whereas the purple area indicates the transition phase from endo- to ecodormancy (determined according to the data shown in Figure 1 in the main text of the manuscript). Mean and median temperatures were calculated between the sampling dates. Example: The weekly mean shown for October 6th 2021 was calculated based on hourly temperature from September 29th 2021, 12 p.m. to October 6th 2021, 11 p.m. For the experimental period of 2019-2020, in which the sampling started in November 24th, the mean and median temperatures prior to November 24th were calculated for each calendar week.
