# Supplementary material for: Identifying indicators of apple bud dormancy status by exposure to artificial forcing conditions
Source: Tree Physiol. 2024 Aug 31;44(10):tpae112. doi: 10.1093/treephys/tpae112 (PMC11447376; doi:10.1093/treephys/tpae112)
Supplement: Suppl_Fig_S5_tpae112 [file suppl_fig_s5_tpae112.pdf]

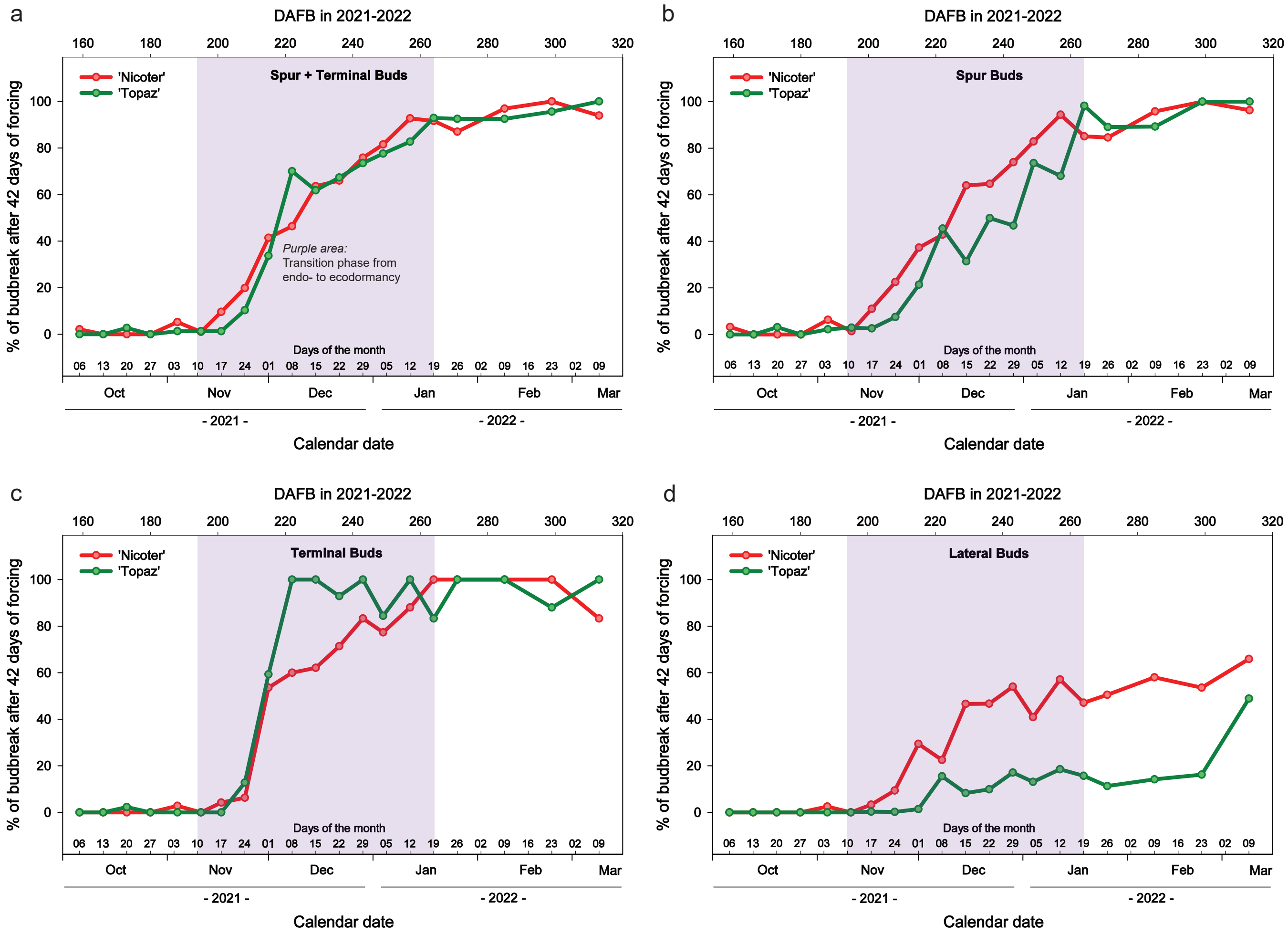

Suppl. Figure 5. Budbreak percentages of terminal, spur, and lateral buds on apple branches sampled from ‘Nicoter’ and ‘Topaz’ trees grown in the orchard in 2021-2022. The branches were kept under budbreak forcing conditions for 42 days weeks.
