# Supplementary material for: Identifying indicators of apple bud dormancy status by exposure to artificial forcing conditions
Source: Tree Physiol. 2024 Aug 31;44(10):tpae112. doi: 10.1093/treephys/tpae112 (PMC11447376; doi:10.1093/treephys/tpae112)
Supplement: Suppl_Fig_S6_tpae112 [file suppl_fig_s6_tpae112.pdf]

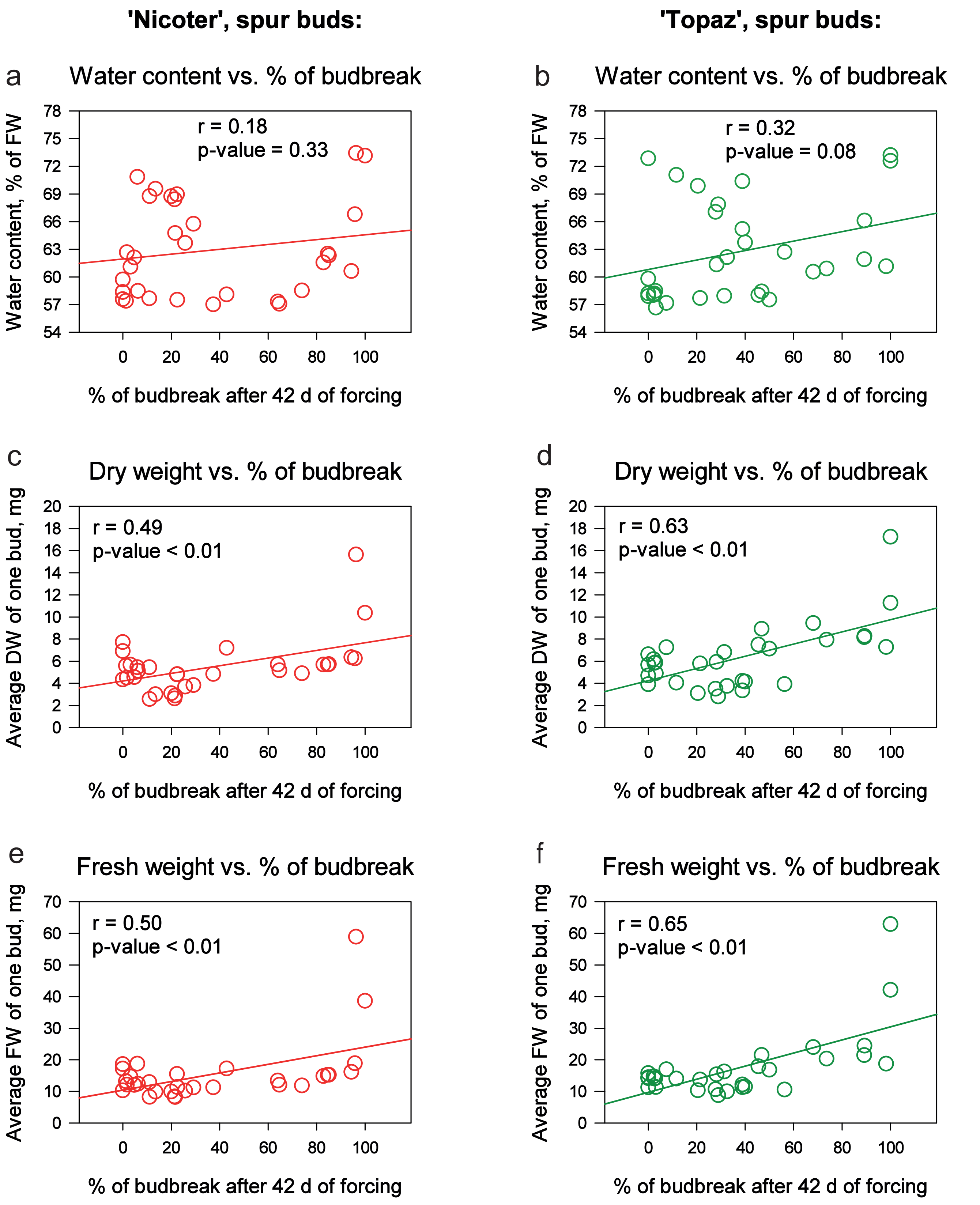

Suppl. Figure 6. Pearson correlation for budbreak percentages vs. water content (a, b), dry weight (c, d) and fresh weight (e, f) of spur buds sampled from ‘Nicoter’ (a, c, e) and ‘Topaz’ (b, d, f).

Each plot contains a regression line along with the information on Pearson correlation (r) and p-value.
