# Supplementary material for: Identifying indicators of apple bud dormancy status by exposure to artificial forcing conditions
Source: Tree Physiol. 2024 Aug 31;44(10):tpae112. doi: 10.1093/treephys/tpae112 (PMC11447376; doi:10.1093/treephys/tpae112)
Supplement: Suppl_Fig_S8_tpae112 [file suppl_fig_s8_tpae112.pdf]

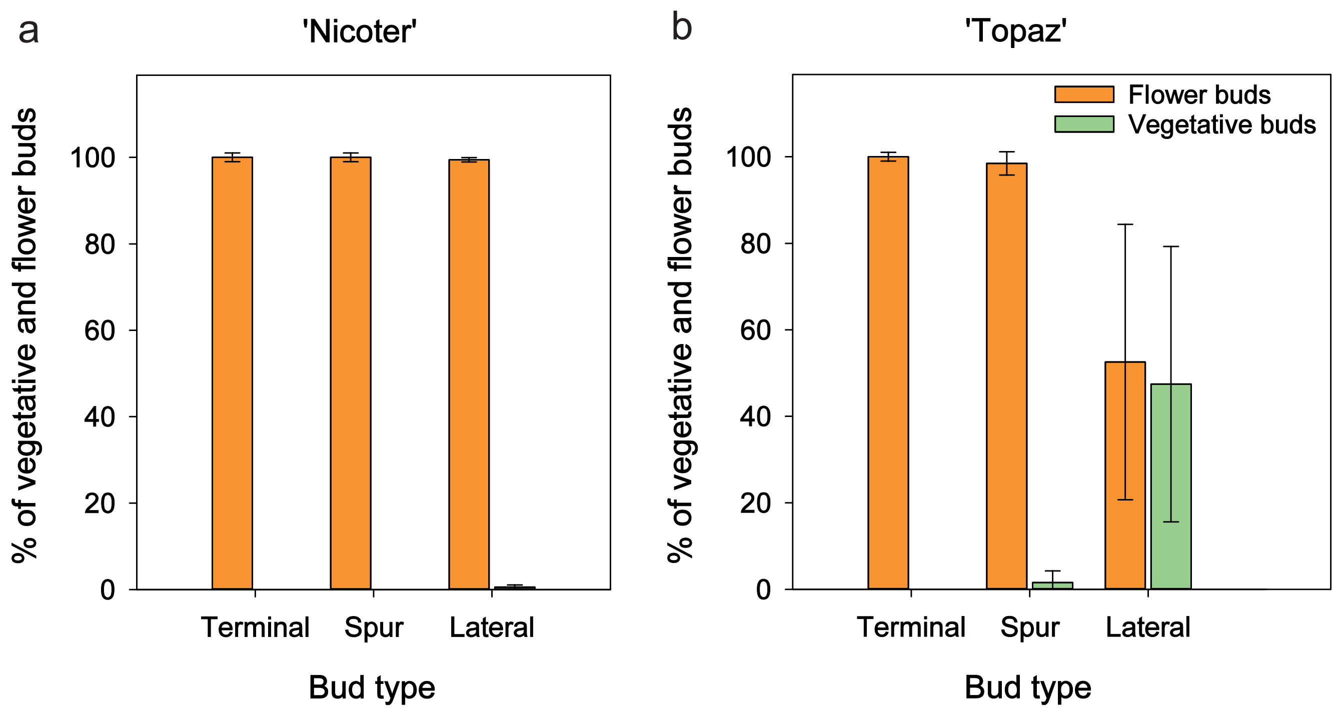

Suppl. Figure 8. Proportion of flower and vegetative buds on branches of ‘Nicoter’ (a) and ‘Topaz’ (b) during ecodormancy (26 January 2022 - 23 February 2022).

The proportions were calculated using three sampling dates as replicates (26 January, 9 February, and 23 February).This data do not reflect the absolute numbers of buds on the branches. The figures only show the proportions of flower-to-vegetative buds for those buds (terminal, spur, and lateral, separately) that reached the stage of budbreak after 42 days of forcing.
