# Supplementary material for: Identifying indicators of apple bud dormancy status by exposure to artificial forcing conditions
Source: Tree Physiol. 2024 Aug 31;44(10):tpae112. doi: 10.1093/treephys/tpae112 (PMC11447376; doi:10.1093/treephys/tpae112)
Supplement: Suppl_Fig_S9_tpae112 [file suppl_fig_s9_tpae112.pdf]

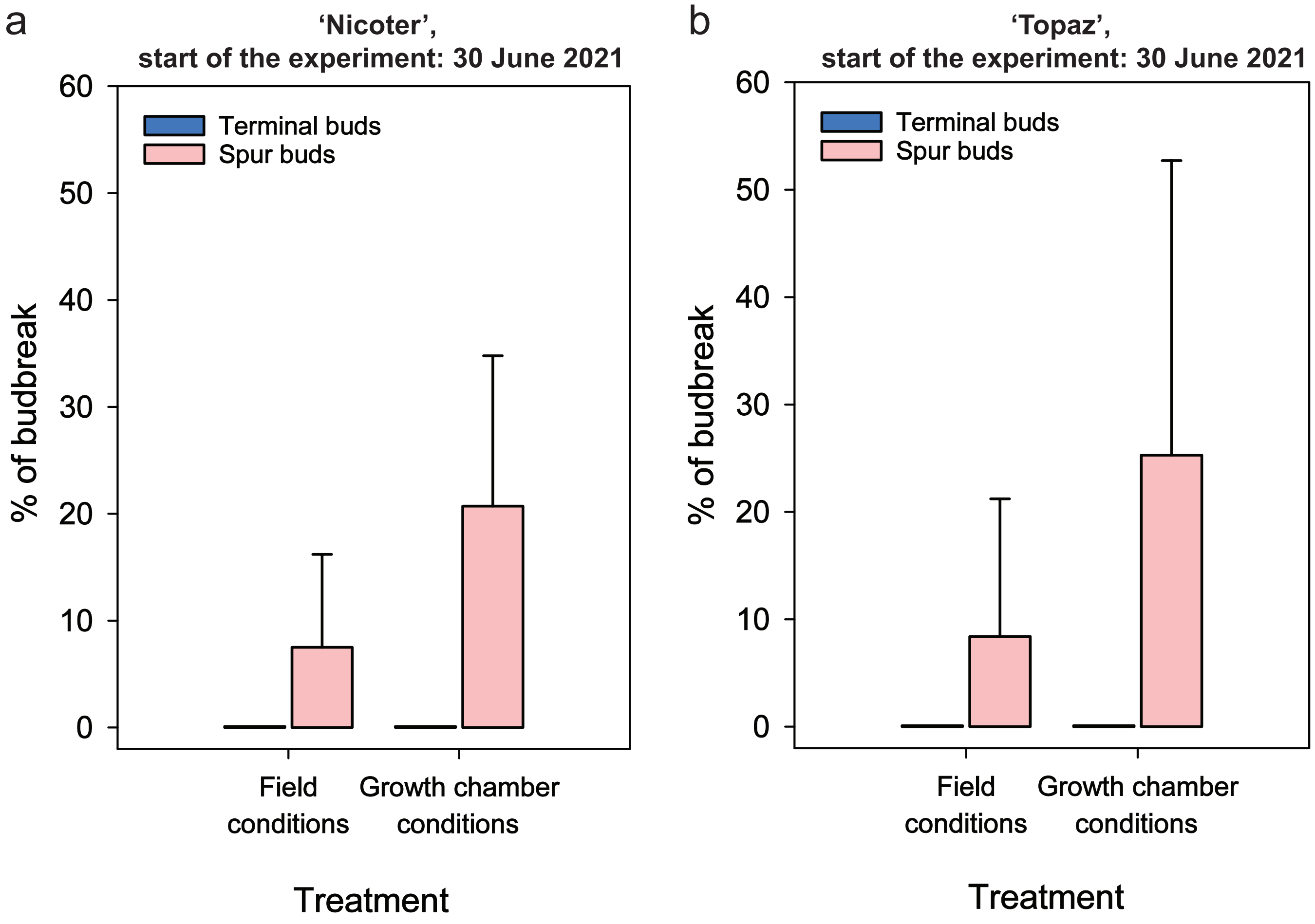

Suppl. Figure 9. Budbreak percentages of terminal and spur buds on defoliated apple branches of 'Nicoter' (a) and 'Topaz' (b) in the orchard (attached branches) and under budbreak forcing conditions (detached branches).

The experiment began on June 30th, 2021. The buds that reached the stage of budbreak were counted after 42 days.
